# Heat shock factor 1, an inhibitor of non-homologous end joining repair

**DOI:** 10.18632/oncotarget.5073

**Published:** 2015-08-24

**Authors:** Ga-Young Kang, Eun-Ho Kim, Hae-June Lee, Na-Yeon Gil, Hyuk-Jin Cha, Yun-Sil Lee

**Affiliations:** ^1^ Graduate School of Pharmaceutical Sciences, Ewha Womans University, Seoul 120–750, Korea; ^2^ Division of Radiation Effects, Korea Institute of Radiological and Medical Sciences, Seoul 139–706, Korea; ^3^ College of Natural Sciences, Department of Life Sciences, Sogang University, Seoul 121–742, Korea

**Keywords:** HSF1, Ku70, Ku86, NHEJ Repair, cellular carcinogenesis

## Abstract

A novel role for HSF1 as an inhibitor of non-homologous end joining (NHEJ) repair activity was identified. HSF1 interacted directly with both of the N-terminal sequences of the Ku70 and Ku86 proteins, which inhibited the endogenous heterodimeric interaction between Ku70 and Ku86. The blocking of the Ku70 and Ku86 interaction by HSF1 induced defective NHEJ repair activity and ultimately activated genomic instability after ionizing radiation (IR), which was similar to effects seen in Ku70 or Ku80 knockout cells. The binding activity between HSF1 and Ku70 or Ku86 was dependent on DNA damage response such as IR exposure, but not on the heat shock mediated transcriptional activation of HSF1. Moreover, the posttranslational modification such as phosphorylation, acetylation and sumoylation of HSF1 did not alter the binding activities of HSF1-Ku70 or HSF1-Ku86. Furthermore, the defect in DNA repair activity by HSF1 was observed regardless of p53 status. Rat mammary tumors derived using dimethylbenz(a)anthracence revealed that high levels of HSF1 expression which correlate with aggressive malignancy, interfered with the binding of Ku70-Ku80. This data suggests that HSF1 interacts with both Ku70 and Ku86 to induce defective NHEJ repair activity and genomic instability, which in turn suggests a novel mechanism of HSF1-mediated cellular carcinogenesis.

## INTRODUCTION

The heat shock factor (HSF)1 activation is critical for maintaining homeostasis of the proteomes of cells and is mediated in large part by increased expression of classical heat shock proteins (HSP) such as HSP27, HSP70, and HSP90 [1]. The HSF1-mediated stress response and the activity of specific HSPs have both been implicated in protecting organisms from a broad range of pathophysiological conditions, including thermal injury, ischemia/reperfusion, and chemotherapeutic agents/ionizing radiation (IR) [2–4]. Much less is known about the role of HSF1 in cancer. It has long been noted that HSP levels increase in a wide range of tumor types [5]. However, the effect of HSF1 activation goes far beyond these chaperones. It helps coordinate a range of fundamental cellular processes that are important to the fitness of malignant cells, including cell cycle control, ribosome biogenesis, protein translation, and glucose metabolism [6, 7].

As a result, HSF1 both facilitates initial oncogenic transformation and maintains the malignant phenotype of established cancer cell lines driven by a wide range of mutations. In mice and in cell culture, genetic ablation of *hsf1* expression potently impairs tumorigenesis and cellular transformation driven by oncogenic activation or tumor suppressor loss [6]. There is controversial evidence that HSF1-mediated carcinogenic effects are dependent on the transcriptional effect on HSP gene expression since elevated expression of one or more of the major HSP classes has been documented in many types of cancers over the years [8]. However, constitutive activation of HSF1 does not fully explain HSP overexpression in cancer cells, because genetic knockdown of HSF1 fails to reduce HSP levels in many cancer cell lines to the normal basal levels seen in non-transformed cells [9]. Moreover, HSP reduction in tumors is unlikely to provide the best surrogate endpoint for monitoring the efficacy of HSF1 inhibitors in clinical trials [9] and some data indicated the possible functions of HSF1 itself, independent of its transcriptional activities [10].

DNA double-strand breaks (DSBs) arise from normal cellular processes, as well as from exogenous sources, such as IR or other forms of genotoxic stress. DNA DSBs are repaired by either homologous recombination (HR) or non-homologous end joining (NHEJ). The faster and more accurate of these repair pathways, DNA-PK dependent NHEJ, is mediated by Ku, DNA-PKcs and Ligase IV [11]. Correct handling of DNA damage is essential for a cell's survival. Cell lines have been observed to inaccurately repair 20% to 25% of their DSBs, depending on whether the breaks are simple or complex [12]. This faulty repair, potentially as a result of the error prone nature of NHEJ [12, 13], can lead to genomic instability, which in turn can lead to onset of cancer [14], either directly in the affected cell or in its progeny [15].

There are some data suggesting the relationship between HSF1 and DNA repair process. HSF1 was reported to bind selectively *in vitro* to Ku protein [16] and the cells with no HSF1 showed a reduced capacity to repair IR-induced DSB [17]. HSF1 is also suggested as a candidate pioneer transcription factor during DNA replication and repair by replication protein A-HSF1 binding [18]. However, the exact roles of HSF1 in the DNA repair pathway still are not known.

Previously, we reported that Plk-mediated HSF1 phosphorylation affect the metaphase to anaphase transition and produce aneuploidy in functional p53-defective cells through a mechanism independent of HSF1 transcriptional activity [10, 19]. Here we identified another novel role for HSF1 as an inhibitor of NHEJ repair activity through blocking the binding of heterodimer Ku70 and Ku86 and ultimately inducing genomic instability.

## RESULTS

### HSF1 inhibits damage repair activity

**Figure 1:**
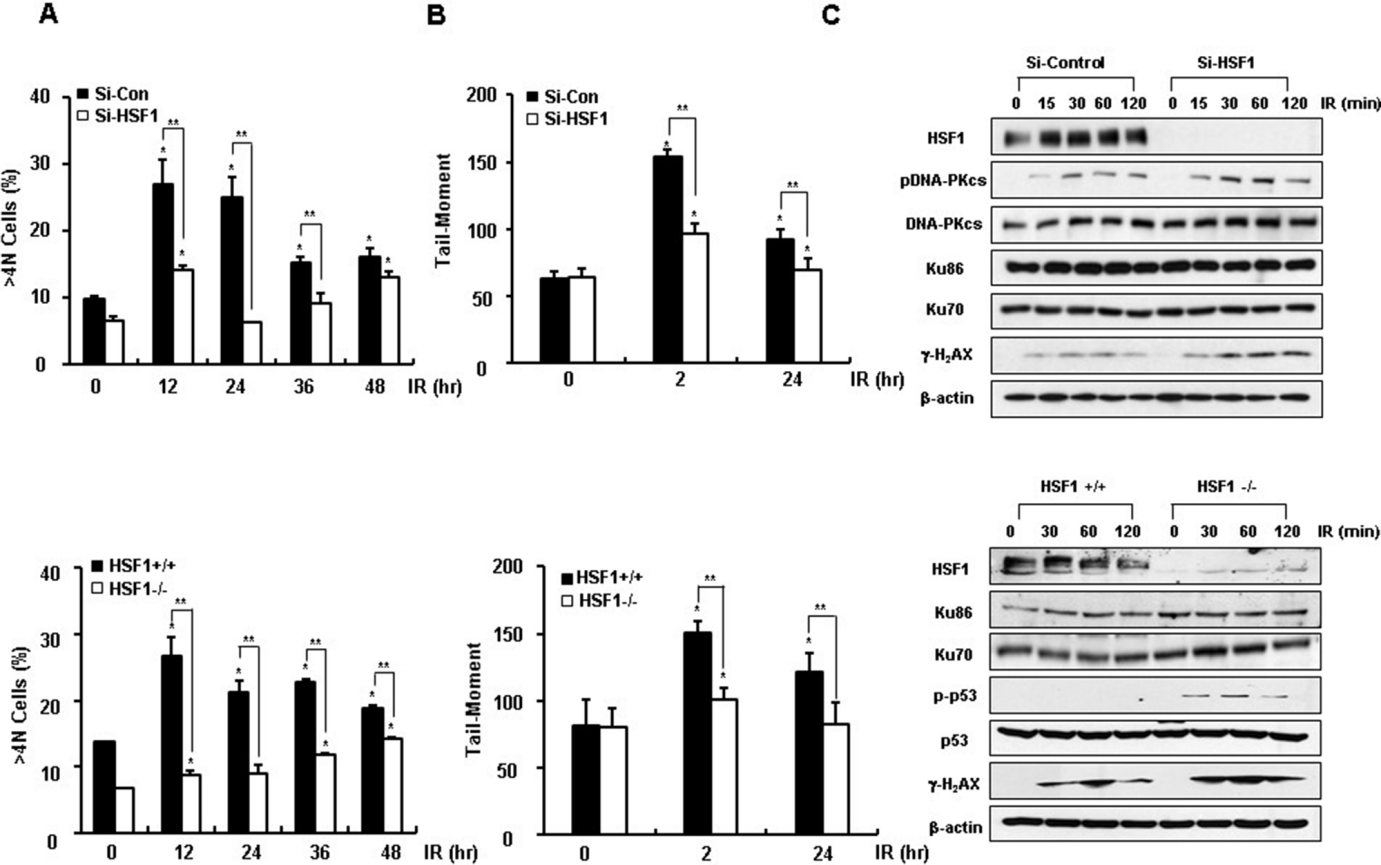
HSF1 inhibits damage repair activity **A.** Si-HSF1 (30nM) transfected HOS cells and HSF1 MEF (HSF1+/+ and HSF1−/−) cells were harvested at the indicated time points following exposed to a dose of 5 and 10 Gy IR. The percentage of the DNA content of cells was measured by flow cytometry. **B.** DSBs were determined through use of the Comet assay. **C.** Western blotting or immunoblotting of Si-HSF1 transfected HOS and HSF1 MEF (HSF1+/+ and HSF1−/−) cell extracts was conducted at the indicated time points following treatment of cells with an IR dose of 5 and 10 Gy. Each data point represents the mean ± SE of three experiments. Significantly different from the corresponding control cells at *p < 0.05. **p < 0.01.

Since our HSF1 binding partners screening assay revealed that Ku70 and Ku86 were the binding partners for HSF1, we examined damage responses after IR in cells with or without HSF1. RNA interference against HSF1 (Si-HSF1) in HOS cells (p53 defective and high HSF1 expressing cells) resulted in decreased aneuploidy production and Comet tail moments in IR-treated cells. HSF1 knockout MEF cells (HSF1−/−, p53 wild type and high HSF1 expressing cells) also showed similar effects (Figure 1A and 1B). When DNA damage repair proteins such as DNA-PKcs, p53, and γ-H_2_AX were examined, HSF1 knockdown cells showed increased phosphorylation of these proteins (Figure 1C). HSF1−/− cells showed increased cell death in response to IR relative to wild-type cells (HSF1+/+), when cell death and cleavage of caspase-3 or PARP1 were evaluated (Supplementary Figure 1). Consequently, this suggested that HSF1 is involved in a defective DNA repair pathway and results in an accumulation of DNA strand breaks and aneuploidy in response to IR, as well as in protection from IR-induced cell death.

### HSF1 interacts with both Ku70 and Ku86

**Figure 2:**
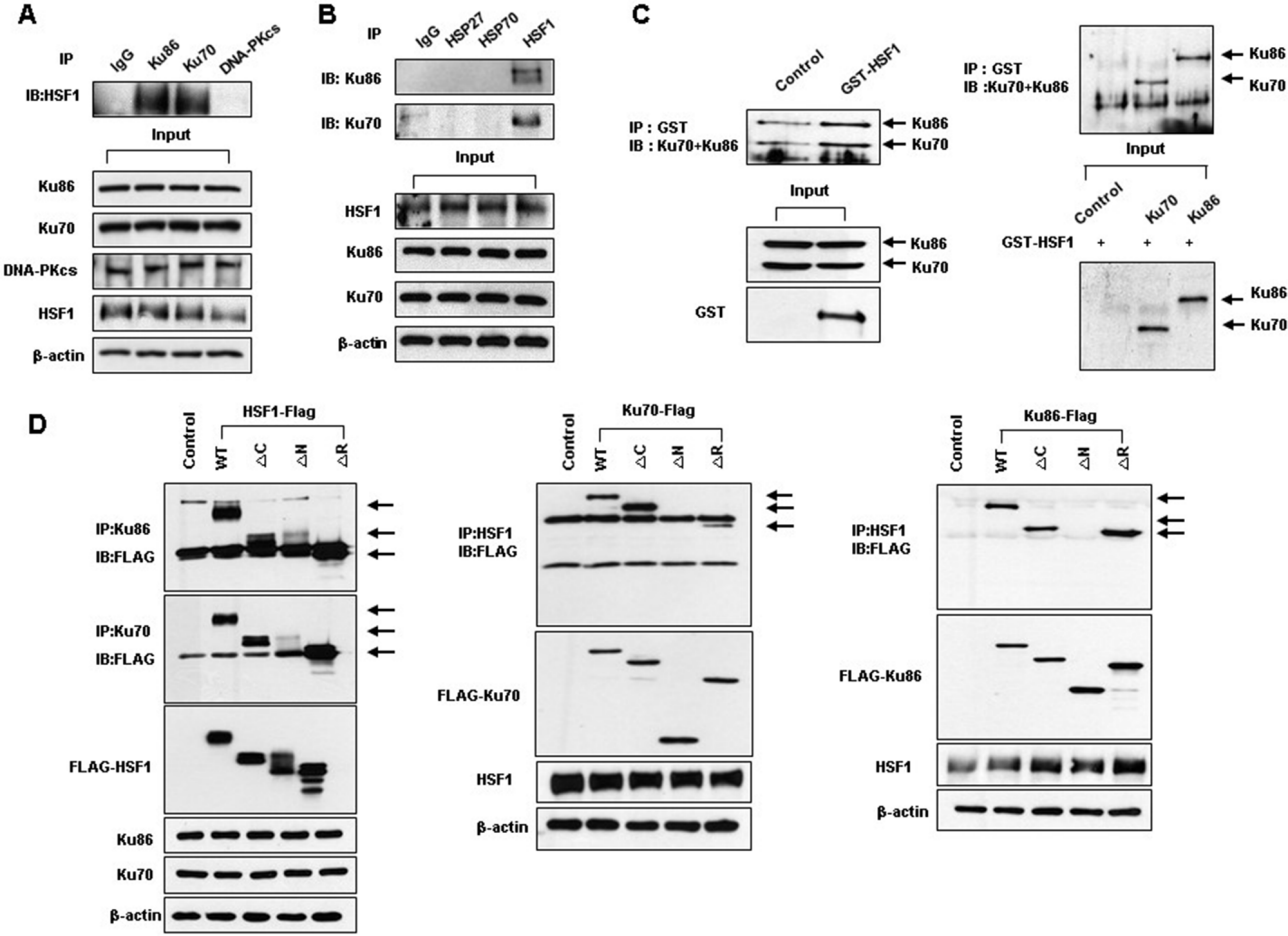
HSF1 interacts with both Ku70 and Ku86 **A, B.** Western blotting was conducted following immunoprecipitation using HOS cell extracts. **C.** Glutathione-*S* transferase (GST) pull-down assays were performed by mixing GST-HSF1 fusion proteins (left) and *in vitro* translated Ku70/Ku86 proteins were incubated with GST-HSF1 and immunoprecipitates were subjected to an *in vitro* binding assay (right). **D.** Various deletion constructs of Flag-tagged HSF1 along with Ku70 and Ku86 constructs were transiently transfected into HEK298T cells. Western blotting was performed following immunoprecipitation using cell extracts.

DNA-PK is composed of two components: a 460 kDa catalytic subunit and a 70- and 86-kDa heterodimeric regulatory component, also known as the Ku protein [20–23]. HSF1 bound specifically to each of two components of DNA-PK (Ku70 and Ku86); however, HSF1 did not bind the catalytic subunit of DNA-PK (DNA-PKcs) (Figure 2A). Moreover, neither HSP27 nor HSP70 interacted with either Ku70 or Ku86 (Figure 2B), suggesting that HSF1-mediated defective repair activity and aneuploidy were not dependent on expression of HSPs which are transcriptional products of HSF1. GST-HSF1 fusion proteins were used as bait to study the interaction between HSF1 and endogenous Ku70 or Ku86 in HOS cells, and GST-pull-down assays were performed to further characterize this interaction. HSF1 directly interacted with both Ku70 and Ku86 in HOS cells (Figure 2C, left). An IP and an *in vitro* translation assay showed the direct interaction between HSF1 and both Ku70 and Ku86 (Figure 2C, right). To identify the specific domain within HSF1 that is required for binding to Ku proteins, we performed studies with deletion mutants of the HSF1 and Ku proteins (Supplementary Figure 2A). The wild-type HSF1 (WT) and C-terminal domain deleted mutant (ΔC) showed approximately equal binding activity, whereas the N-terminal domain deleted mutant of HSF1 (ΔN) was severely reduced in binding activity (Figure 2D, left). The N-terminal domain of HSF1 was determined to be the domain that interacts with Ku70 or Ku86. In the case of Ku70 and Ku86 were found to be the domains that interacted with HSF1 (Figure 2D, middle and right), suggesting that the N-terminal domain of HSF1 can interact with the N-terminal domains of Ku70 or Ku86. Other mammalian isoforms of HSF, such as HSF2 and HSF4, did not interact with either Ku70 or Ku86 (Supplementary Figure 2B).

### HSF1 inhibits heterodimeric binding activity between Ku70 and Ku86

**Figure 3:**
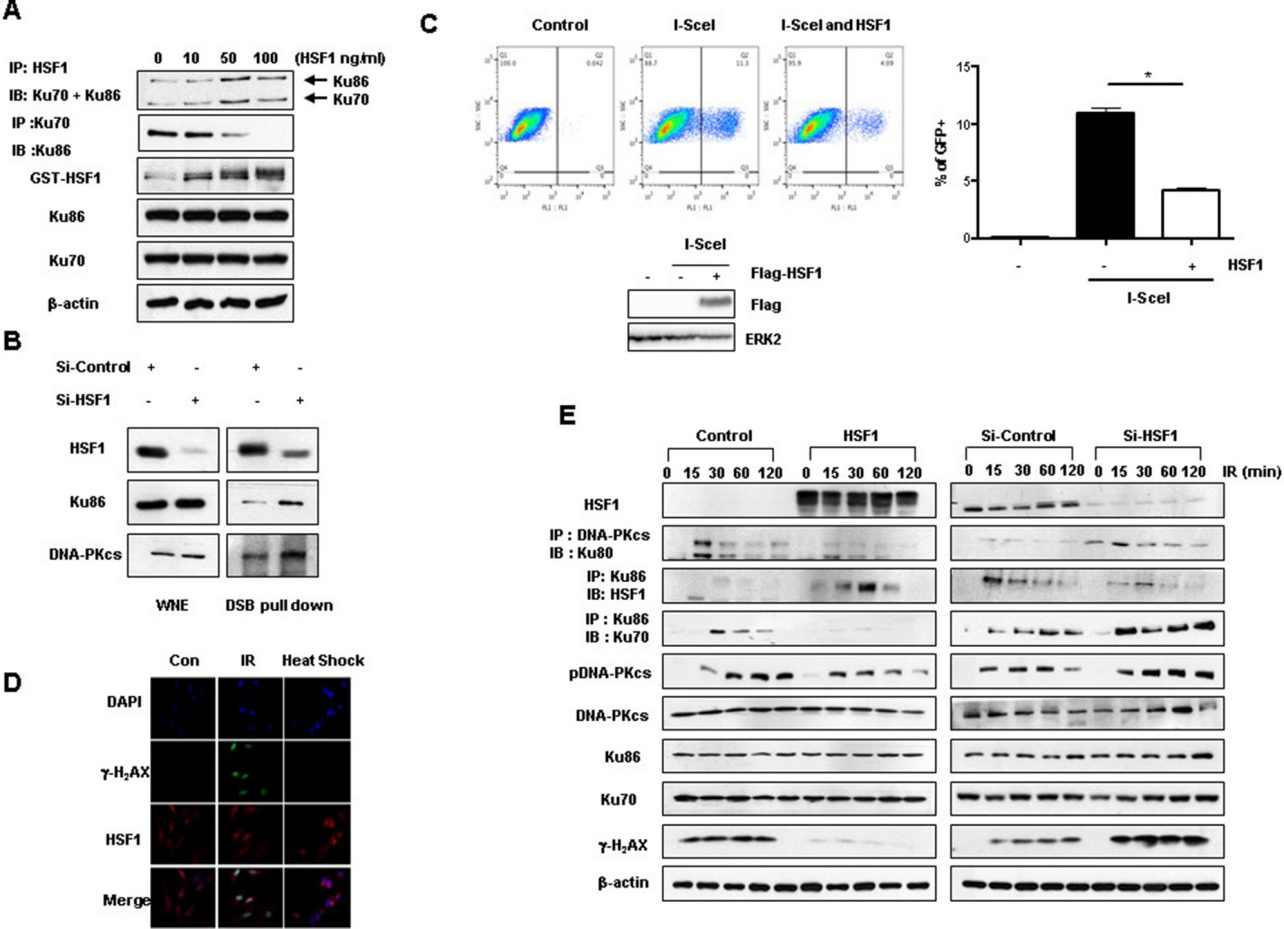
HSF1 inhibits binding activity between Ku70 and Ku86 **A.** Purified recombinant HSF1-GST proteins (0, 10, 50, and 100 ng/ml) were added to HOS cell extracts. After 16 hrs, western blotting was performed following immunoprecipitation using cell extracts. **B.** Si-Control or Si-HSF1 (30 nM) transiently transfected into HOS cells. The protein levels of HSF1, Ku86 and DNA-PKcs in the dsDNA pull-down lysates, as well as in complete whole-nuclear extracts (WNE), were analyzed. **C.** GFP+ cells with mock or HSF1 expression in U2OS cells stably expressing EJ5-GFP were determined by FACS analysis. GFP+ cells were graphically presented. HSF1 expression level was determined by immunoblotting with Flag. ERK2 was used for equal loading control. *p < 0.05. **D.** HOS cells were irradiated (5 Gy) or heat shock (42°C) for 1 h and stained for HSF1 (red), γ-H_2_AX (green), and DAPI. Cells were analyzed using immunofluorescence microscopy. **E.** HOS cells were transiently transfected with Si-Control or Si-HSF1 (30 nM) and control or HSF1 wild type plasmids. Cells were then exposed to a dose of 5 Gy IR and immunoblotting or immunoprecipitation were performed at the indicated time points.

To examine whether binding between HSF1 and Ku70 or Ku86 affects the endogenous heterodimerization of Ku70 and Ku86, HSF1 protein was added and interaction activity was evaluated using immunoprecipitation. The endogenous interaction between Ku70 and Ku86 was inhibited in a concentration dependent manner by the HSF1 protein, while the interaction between HSF1 and Ku70/Ku86 was increased (Figure 3A). Because the DNA-PK complex, including Ku70 and Ku86, is recruited to DNA DSB sites after DNA damage [24], we next determined whether the interaction of Ku70 or Ku86 with HSF1 occurred at DSB sites. We employed a DSB pull-down assay using exogenously transfected double stranded (ds) oligonucleotides to evaluate the association of the DNA-PK complex and HSF1 with DNA DSBs. Then, we showed that the association of the DNA-PK complexes with DSBs was significantly reduced in a Si-Control HOS nucleus as compared with that in a Si-HSF1 HOS nucleus (Figure 3B). However, overexpression of HSF1 inhibited the association of DNA-PK complexes with DSBs (Supplementary Figure 3). The reporter systems which can distinguish the DSB repair pathways, NHEJ or HR, based on the enhanced green fluorescent protein (GFP) and meganuclease such as I-SceI [25], indicated that HSF1 overexpression reduced NHEJ repair activity (Figure 3C), but not HR repair activity (Supplementary Figure 4). Immunostaining data also suggested that the IR damage sites colocalized with HSF1 in nuclei when γ-H_2_AX and HSF1 were co-stained, while in the case of heat shock response, γ-H_2_AX was not induced (Figure 3D). Next, we performed a kinetic analysis of the binding affinity between HSF1 and the DNA-PK complex. IR increased the binding activity between Ku70 and Ku86, as well as the binding between DNA-PKcs and Ku86. However, HSF1 overexpression inhibited these binding activities, and consequently, the interaction between HSF1 and Ku86 was potentiated. The phosphorylation of DNA-PKcs and γ-H_2_AX expression by IR were decreased remarkably in HSF1 overexpressing cells when compared to control cells. When we examined these phenomena in HSF1 knockdown cells, opposite effects were observed (Figure 3E).

### Knockout of Ku70 or Ku80 results in similar patterns of HSF1 overexpression with defective DNA repair and aneuploidy formation

**Figure 4:**
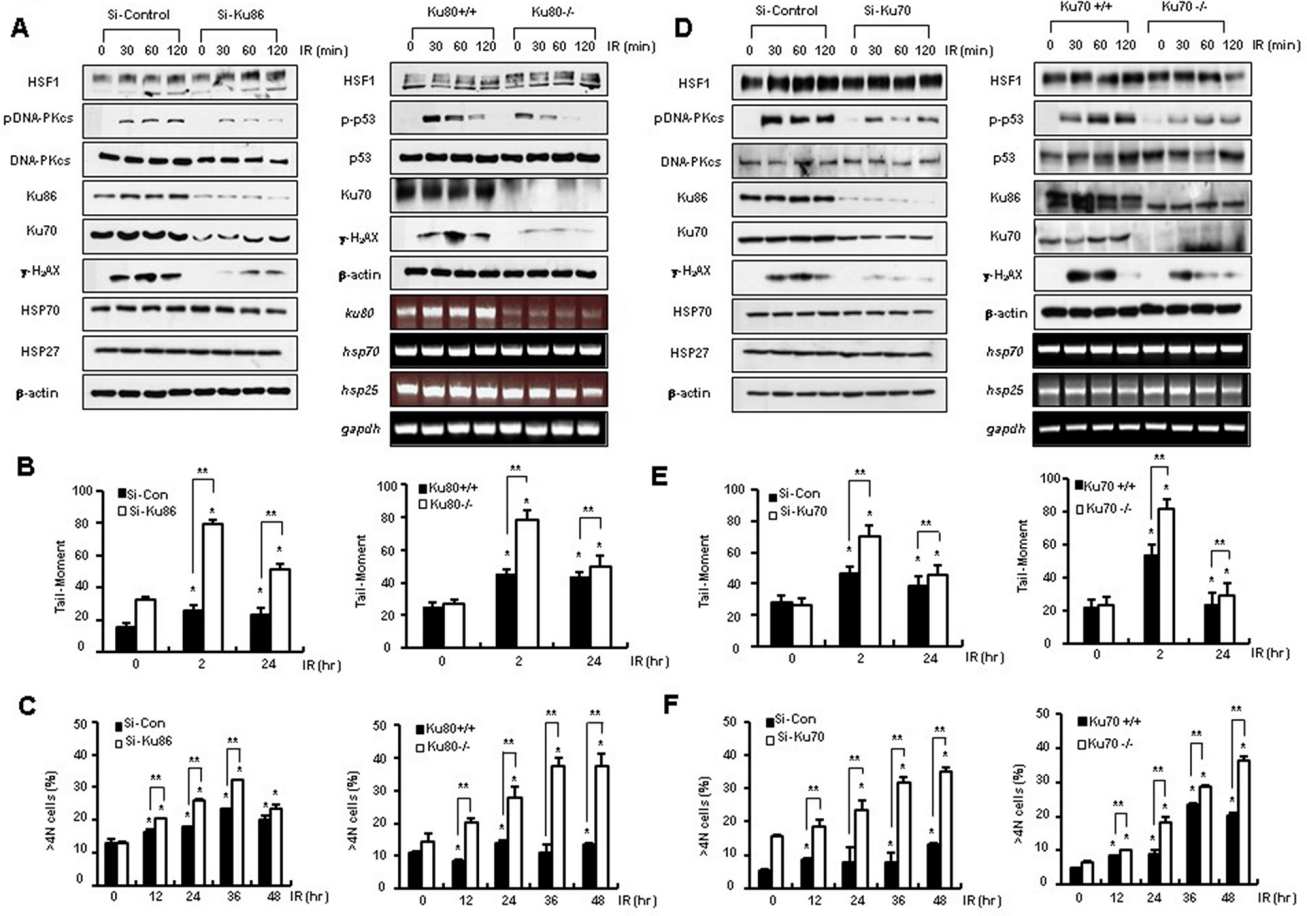
Knockout of Ku70 or Ku80 shows a pattern similar to HSF1 overexpression **A.** Si-Control or Si-Ku86 (30 nM) transfected HOS cells and Ku80 defective cells were exposed to doses of 5 and 10 Gy IR. Western blotting and RT-PCR were performed at the indicated time points. **B, E.** The presence of DSBs was determined using the Comet assay. **C**, **F.** The percentage of the DNA content of cells was measured by flow cytometry. **D.** Si-Control or Si-Ku70 (30 nM) transfected HOS cells and Ku70 knockout cells were exposed to a dose of 5 and 10 Gy IR. Western blotting and RT-PCR were performed at the indicated time points. Each data point represents the mean ± SE of three experiments. Significantly different from the corresponding control cells at *p < 0.05. **p < 0.01.

Since HSF1 interfered with the binding of Ku70 and Ku86 and inhibited the activity of Ku70- or Ku86-mediated repair, the effects of a knockout of Ku70 or Ku80 were examined to determine if the effects were similar to those of HSF1 overexpression. Knockdown of Ku86 in HOS cells and Ku80 knockout cells resulted in an inhibition of expression of γ-H_2_AX and phospho-p53 induced by IR, without any alteration in HSP expression levels (Figure 4A). Increased Comet tail moments and aneuploidy formation by IR were also demonstrated to occur in Ku86 knockdown HOS cells and Ku80 knockout cells (Figure 4B and 4C). Knockdown of Ku70 and Ku70 knockout cells gave similar results (Figure 4D, 4E and 4F). When cell death and cleavage of caspase 3 or PARP1 were evaluated, IR treated Ku80−/− cells showed an increase in cell death compared with that seen in Ku80+/+ cells (Supplementary Figure 5). Therefore, this suggests that the role of HSF1 in defective DNA repair activity and aneuploidy formation by IR, as well as in sensitization of cells to IR-induced cell death, was similar to the effect seen upon knockout of Ku proteins.

### HSF1-mediated defective repair activity is independent of its transcriptional activity

**Figure 5:**
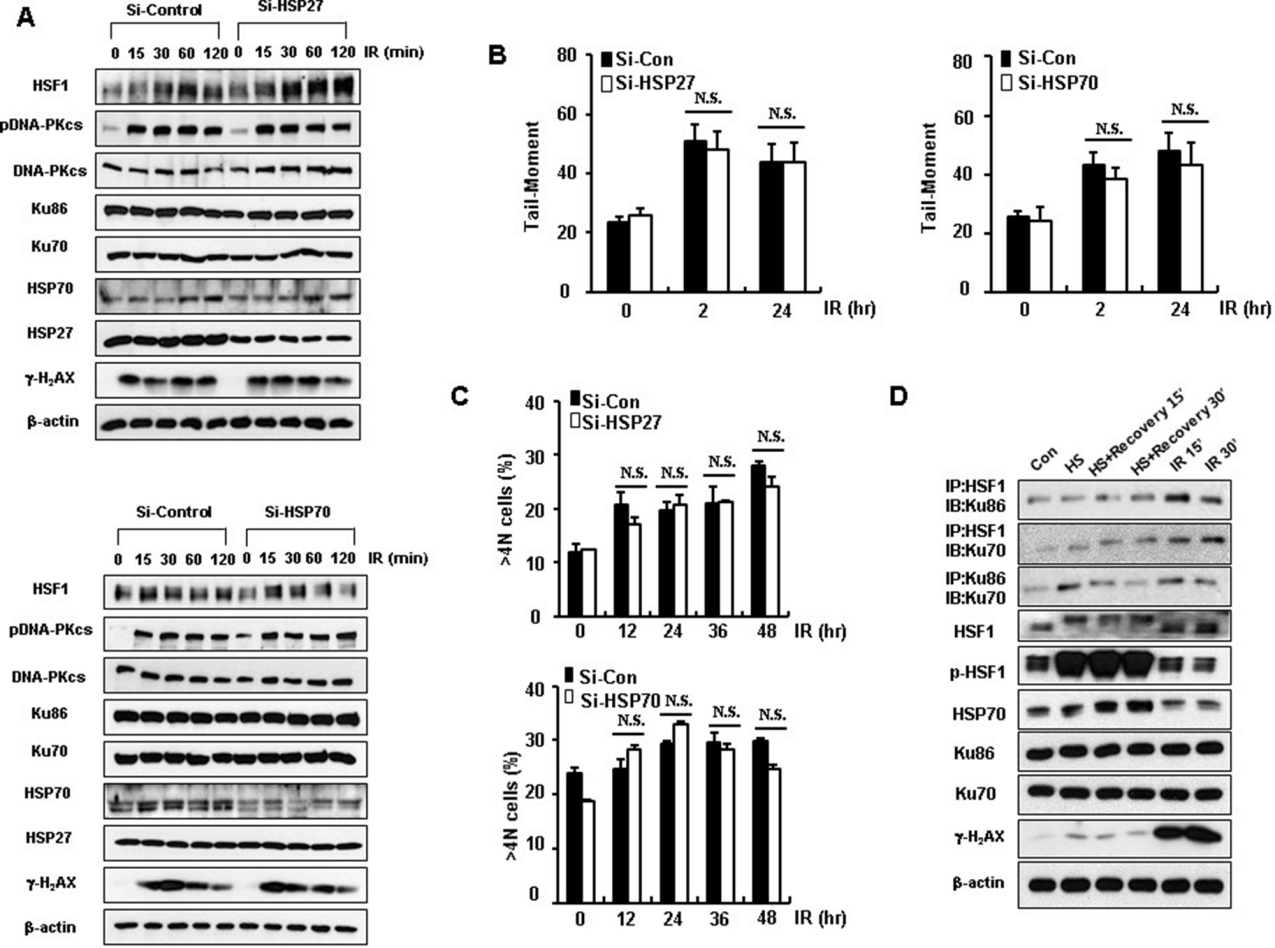
HSF1-mediated defective repair activity is independent of its transcriptional activity **A.** Western blotting or immunoblotting of Si-HSP27 or Si-HSP70 transfected HOS cells extracts was conducted at the indicated time points following treatment of cells with an IR dose of 5 Gy. The results represent one of three independent experiments. **B.** DSBs were determined through use of the Comet assay. **C.** The percentage of the DNA content of cells was measured by flow cytometry. Each data point represents the mean ± SE of three experiments *vs* control. **D.** HOS cells were heat shocked (HS) for 30 min at 42°C followed by indicated recovery times or exposed IR (5 Gy). Total proteins were analyzed for Western blotting or immunoprecipitation.

Since HSF1 is a well-known transcription factor for HSPs, HSF1-mediated defective repair activity and aneuploidy production may be dependent upon its transcriptional activity. When knockdown of HSP27 or HSP70 was performed in HOS cells, DNA repair activities, such as production of Comet tails or γ-H_2_AX formation in response to IR, were unchanged (Figure 5A and 5B). Similarly, aneuploidy formation as a result of IR was not altered by knockdown of HSP27 or HSP70 (Figure 5C). Even though HSF1−/− cells showed reduced expression of HSP27 or HSP70 when compared to HSF1+/+ cells, in HOS cells knockdown or overexpression of HSF1 did not affect the expression levels of HSP27 or HSP70 (Supplementary Figure 6A and 6B). When it was examined if binding activity between HSF1 and Ku70 or Ku86 was dependent on heat shock (HS)-mediated transcriptional activity of HSF1 in HOS (Figure 5D) and NCI-H460 (Supplementary Figure 6C) cells, 15 min or 30 min recovery after HS dramatically induced HSP70 in accompanied with the increased phosphorylation of HSF1 at Ser326, suggesting transcriptional activation of HSF1. HS did not alter the binding activity of HSF1-Ku70 or HSF1-Ku86. However, IR exposure that dominantly showed the increased expression of γ-H_2_AX, increased the binding activity of HSF1-Ku70 or HSF1-Ku86, even though transcriptional activation of HSF1 was not observed, suggesting that binding activity between HSF1 and Ku70 or Ku86 was only occurred by DNA damage response, but not by the transcriptional activation of HSF1.

### Posttranslational modification of HSF1 and cellular p53 status do not affect the HSF1-Ku70 or HSF1-Ku86 interactions

**Figure 6:**
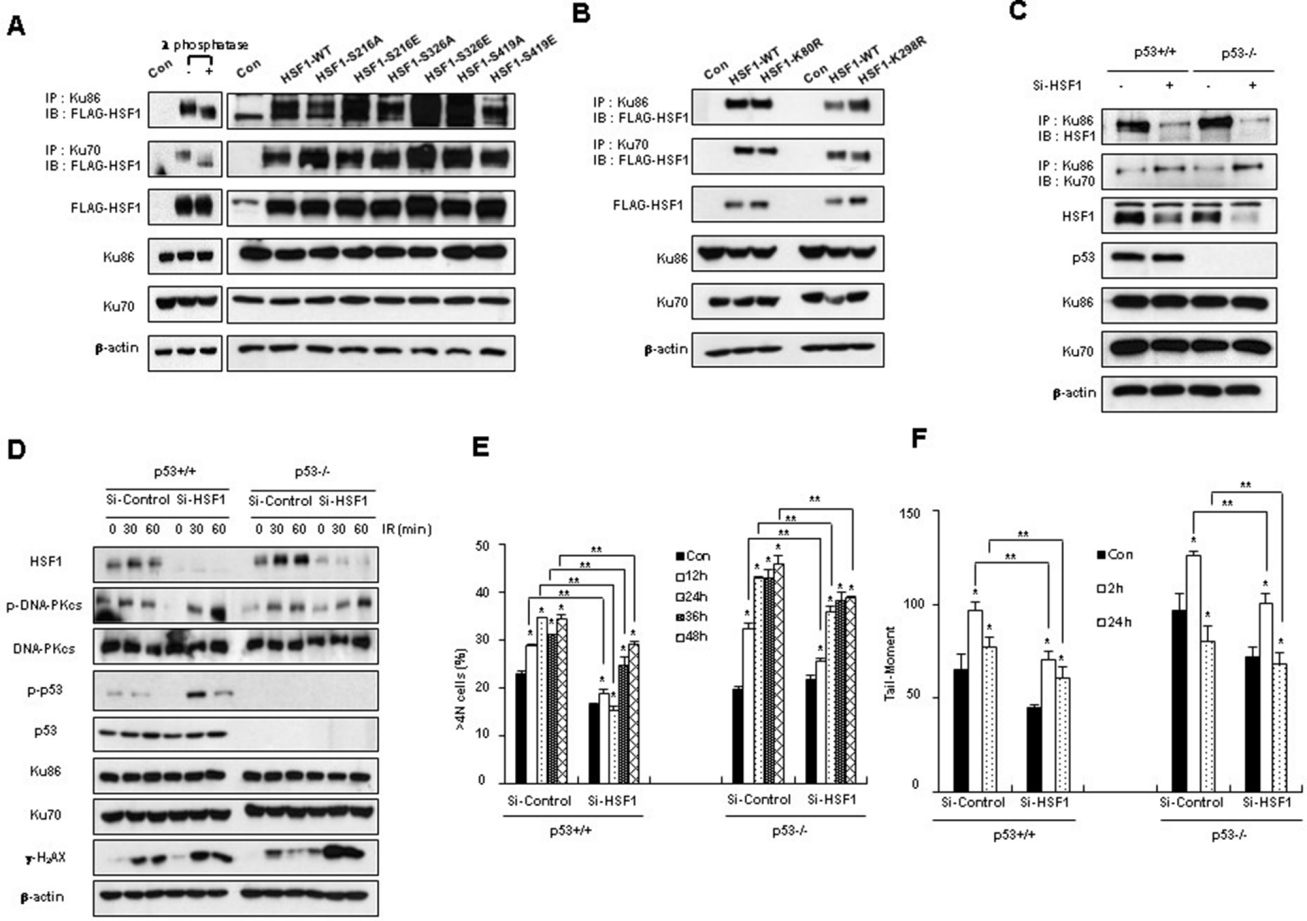
Posttranslational modification of HSF1 and cellular p53 status does not affect the interaction of HSF1-Ku70 or HSF1-Ku86 **A.** Ku70 or Ku86 immunoprecipitations were performed, and each immunoprecipitation reaction mixture was either mock treated (−) or treated with lambda phosphatase (+) (left). Control and Flag-HSF1 phospho-mutant constructs were transfected into HEK293T cells and western blotting or immunoblotting was conducted following immunoprecipitation on cell extracts (right). **B.** Control and Flag-HSF1 acetylation-defective mutant or HSF1 sumoylation mutant constructs were transfected into HEK293T cells and Western blotting was performed following immunoprecipitation on cell extracts. **C, D.** Si-Control or Si-HSF1 transfected HCT116 (p53+/+ and p53−/−) cells were exposed to a dose of 5 Gy IR. Western blotting was performed following immunoprecipitation using cell extracts. **E.** The percentage of the DNA content of cells was measured. **F.** The presence of DSBs was determined using the Comet assay. Each data point represents the mean ± SE of three experiments. Significantly different from the corresponding control cells at *p < 0.05. **p < 0.05.

Since HSF1 interacts with Ku70 and Ku86 in the nucleus and posttranslational modification of HSF1 such as phosphorylation, acetylation or sumoylation is reported to be able to modify the DNA binding activity of HSF1 [26–28], several point mutants of phosphorylation, acetylation and sumoylation sites were prepared and IP experiments were performed. The HSF1 phosphorylation including ser216, ser326 and ser419, HSF1 acetylation including lys80 or HSF1 sumoylation including lys298, did not affect binding between HSF1 and Ku70 or Ku86 (Figure 6A and 6B).

Since p53 regulates NHEJ repair [29–31], the interaction of HSF1 with Ku70 or Ku86 was examined in cells with different p53 statuses such as HCT116 (p53+/+ and p53−/−) cells. The interaction between HSF1 and Ku70 or Ku86 and Ku86 did not differ according to p53 status (Figure 6C). Likewise, γ-H_2_AX and p53 downstream signaling which were different according to the HSF1 status, were not affected by differences in p53 status. HSF1 knockdown facilitated events downstream of p53 such as phosphorylation of DNA-PKcs and γ-H_2_AX formation even in p53−/− cells (Figure 6D). Increased formation of aneuploidy and Comet tail moments by IR were inhibited by knockdown of HSF1. p53 knockout cells showed an more increase in aneuploidy and comet tail moments than p53+/+ cells, while HSF1 knockdown still inhibited these phenomena (Figure 6E and 6F).

### HSF1 expression correlates with aggressive malignancy and negative binding activity of Ku70-Ku80 in rat mammary tumors

**Figure 7:**
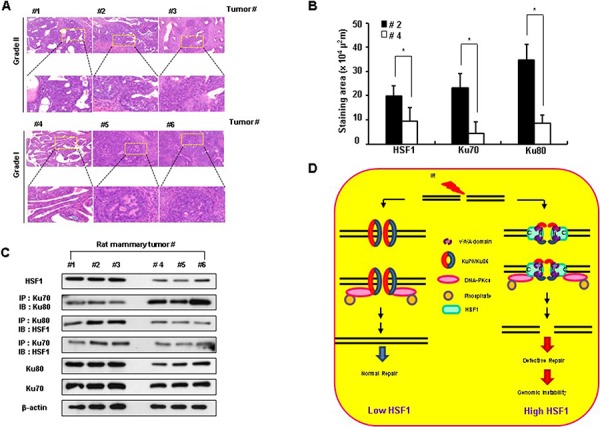
HSF1 expression correlates with aggressive malignancy in rat mammary tumors H&E staining **A.** and staining for HSF1, Ku70 and Ku80 **B.** in rat mammary adenocarcinomas. Statistical significance from rat mammary tumors was set to *p < 0.05. **p < 0.01. **C.** Western blotting was performed following immunoprecipitation using rat mammary tumor tissue extracts. **D.** Hypothetical scheme that HSF1induces defective repair activity by interfering with the interaction between Ku70 and Ku86.

To elucidate the physiological role of HSF1 in tumorigenesis, we examined the relationship between HSF1 expression and tumor malignancy in spontaneously induced rat mammary tumors. Rat mammary tumors were induced by DMBA [32] and these tumors were all malignant adenocarcinomas. High HSF1 expressing mammary tumors were determined to be aggressive malignancies (Grade II) when their morphology was evaluated using the parameters of Hilf *et al*. [33], while low HSF1 expressing tumors showed little malignancy (Grade I) (Figure 7A and 7B, Supplementary Figure 7). Moreover, high HSF1 expressing tumors showed low interaction activity between Ku70 and Ku80, while the binding activity of HSF1 and Ku70 or HSF1 and Ku80 was high. Tumors expressing low levels of HSF1 showed opposite effects (Figure 7C).

## DISCUSSION

In this paper, aneuploidy, a characteristic of most human cancers, seemed to be activated by HSF1, independent of its transcriptional activity such as HSP expression. Therefore, since the regulation of the major HSPs does not explain the entire range of HSF1 functions, there are questions remaining regarding an alternative mechanism for how HSF1 may be involved in cancer initiation or progression. Our results provide some clues to this. We identified a novel role for HSF1 as an inhibitor of NHEJ repair activity through blocking the binding of Ku70 and Ku86 and ultimately inducing defective DNA repair and genomic instability. HSF1 interacts independently with either Ku70 or Ku86 and inhibits heterodimerization of Ku70 and Ku86. Heterodimerization of Ku70 and Ku86 is a key process in NHEJ repair pathways after IR damage and inhibition of this heterodimerization by HSF1 induces defective repair activity and aneuploidy. Ku70 and Ku86 interact with HSF1 via their N-terminal domains (Von Willebrand A domain, vWA) of Ku70 or Ku86. vWA is a well-studied domain involved in cell adhesion, in extracellular matrix proteins, and in integrin receptors [34]. In Ku70 and Ku86, the region encompassing the vWA domain is the determinant of heterodimerization, which is consistent with the role of vWA in protein-protein interactions [34–36]. In addition, some of the numerous protein-protein interactions demonstrated for the eukaryotic Ku proteins, in addition to heterodimerization, likely depend on the vWA domains of Ku70 and Ku86 [35, 37]. Heterodimerization between Ku70 and Ku80 is essential for DNA DSB repair and is also important in activating DNA-PK, which is one of the main functions of Ku [38–40]. Therefore, it has been suggested that HSF1 interacts with the vWA domains of both Ku70 and Ku86 and inhibits heterodimerization of Ku70 and Ku86.

As only HSF1, but not HSF2 and HSF4, has been shown to bind to Ku70 and Ku86, this suggests a unique function for HSF1 in DNA repair pathways. Indeed, when we examined phospho-DNA-PKcs, γ-H_2_AX, Comet tail formation, and aneuploidy, HSF1 increased expression of each of these. Moreover, HSF1-mediated defective DNA repair activity is not seen when HSPs such as HSP27 and HSP70 were overexpressed. Moreover, HS-mediated transcriptional activation of HSF1 is not involved in the binding activity between HSF1 and Ku86 or Ku70, suggesting independency from the transcriptional activity of HSF1. Therefore, it is hypothesized that HSF1 has dual roles of transcriptional and non-transcriptional functions. Transcriptional functions of HSF1 such as HSP production induce the cellular survival, while non-transcriptional functions of HSF1 show the defective repair activity and genomic instability in the survived cells.

Spontaneous rat mammary tumors that overexpressed HSF1 that developed in response to DMBA showed aggressive carcinogenesis with inhibition of binding activity between Ku70 and Ku80, suggesting a physiological role for HSF1 in tumor development by inhibition of NHEJ repair activity.

Posttranslational modifications of HSF1 such as phosphorylation, acetylation or sumoylation importantly affect the HSF1 activity [25–27]. However, in the case of NHEJ regulation of HSF1, posttranslational modifications are not involved, even though all the modifications of HSF1 are not examined in this study. We do not know the upstream pathways for HSF1 binding activity with Ku70 or Ku86, however, at least, DNA damage responses after IR may be importantly involved.

Faulty DNA repair, potentially as a result of the error prone nature of NHEJ [12, 13], can lead to genomic instability, which in turn can lead to the onset of cancer [14], either directly in the affected cell or in its progeny [15]. Furthermore, factors traditionally linked to accurate repair, such as Ku, may also be linked to mis-joining of breaks [15]. Therefore, HSF1, which inhibits heterodimerization of Ku70 and Ku86, may have a role in defective NHEJ repair and may be involved in the promotion of genome instability.

In our previous study, we reported that HSF1 phosphorylation by Plk1 in mitosis and prolonged Plk1-mediated HSF1 phosphorylation affect the metaphase to anaphase transition and produce aneuploidy in functional p53 defective cells through a mechanism independent of its transcriptional activity [10, 19]. However, p53 status was not important for HSF1 and Ku binding. This discrepancy suggested that different roles of p53 in regulation of HSF1-mediated mitosis or NHEJ repair process.

In conclusion, we identified a novel function of HSF1 as an inhibitor of NHEJ repair through inhibition of heterodimerization of Ku70 and Ku86, which affects genomic instability and cancer development (Figure 7D). Therefore, interfering with the protein-protein interaction of HSF1 and the vWA domains of Ku70 or Ku86 may be an effective strategy for inhibiting tumorigenesis.

## MATERIALS AND METHODS

### Cell culture

HOS (human osteosarcoma cell) cells was cultured in RPMI medium supplemented with 10% fetal bovine serum and 1% penicillin-streptomycin (Gibco) at 37°C in humidified 5% CO_2_ incubator. HSF1 knockout mouse embryonic fibroblast (HSF1+/+ and HSF1−/− MEF) cells were provided by Dr. Ivor J. Benjamin (University of Utah, Salt Lake City, UT). Ku70 knockout mouse embryonic fibroblast (Ku70+/+ and Ku70−/− MEF) cells were provided by Dr. S. Matsuyama (The University of Western Ontario, Canada). Ku80-defective xrs6 and CHO-K1 cells were provided by Dr. Bernard S. Lopez (Centre National de la Recherche Scientifique/Commissariat à l'Energie Atomique, France). HEK293T (human embryonic kidney cell) cells were cultured in Dulbecco's minimal essential medium (DMEM), supplemented with heat-inactivated 10% fetal bovine serum (FBS) and antibiotics.

### Preparation of HSF1 mutant constructs

A full-length human HSF1 cDNA and deletion constructs of HSF1, Ku70 and Ku86 were cloned into the expression vector p3xFlag-myc-CMVTM-26, which were generated by Cosmo (Cosmo Gentech, Inc). Site-directed mutagenesis was carried out using the QuickChange™ mutagenesis kit (Stratagene). The constructs HSF1-S216A, HSF1-S216E, HSF1-S326A, HSF1-S326E, HSF1-S419A, HSF1-S419E (numbers indicate the phosphorylation sites), HSF1-K80R (number indicates the acetylation site) and HSF1-K298R (number indicates the sumoylation site) were generated using as template wild-type HSF1.

### Antibodies and reagents

Antibodies against HSF1, HSP70, HSP27, DNA-PKcs, Ku86, Ku70, β-actin, GST, HA, Histone H1, and p53 were purchased from Santa Cruz Biotechnology (Santa Cruz, diluted 1:1000). Antibodies against p-p53, p-DNA-PKcs (Ser 2056), γ-H2AX, FLAG, p-HSF1 (Ser 326), and Ku70 (N3H10) were obtained from Cell Signaling Technology, Abcam, Millipore, Sigma-Aldrich, and Thermo Scientific, respectively (diluted 1:1000). Pre-designed siRNAs for human HSF1, HSP27, HSP70, Ku70, Ku86, and a negative control Si-RNA (30 nM) were purchase from Bioneer Corporation. Propidium iodide (PI) and RNase A were purchased from Sigma-Aldrich.

### Cell transfection

Transient transfection of all cell types was carried out using Lipofectamine 2000 (Invitrogen), according to the manufacturer's guidelines.

### *In vitro* translation analysis

*In vitro* transcription/translation of the full-length wild-type Ku70 and Ku86 proteins was performed using the TNT T7 Quick Master Mix kit (Promega) in the presence of [35S] methionine, according to the manufacturer's protocol.

### Cell cycle analysis

For cell cycle analysis, the cells were fixed in 70% ethanol at −20°C for at least 18 h. The fixed cells were washed once with PBS-EDTA and resuspended in 1 ml of PBS. After the addition of 10 μl each of propidium iodide (5mg/ml) and RNase (10 mg/ml), the samples were incubated for 30 min at 37°C and analyzed using a FACScan flow cytometer (BD Biosciences).

### Linear dsDNA-associated protein pull-down assay

Nuclear extracts were isolated for a dsDNA pull-down assay. A biotinylated oligonucleotide (1 kb) generated by PCR amplification of pcDNA3 (5.4 kb) with the biotinylated forward primer 5′-GACTCTCAGTACAATCTGCTCTGA-3′ and the reverse primer 5′-AGCTCTAGCATTTAGGTGACACT-3′, was immobilized on streptavidin beads (Sigma). Immobilized DNA was mixed with nuclear extracts and incubated for 3 h at 4°C. The beads were washed with buffer D (10 mM Tris, pH 7.6, and 100 mM NaCl) and boiled for 5 min in 2 × SDS sample buffer. Double-stranded DNA-associated proteins were analyzed by SDS-PAGE and visualized with enhanced chemiluminescence detection (ECL, Amersham).

### Immunoblotting and immunoprecipitation

For immunoblotting, cells were lysed with radioimmune precipitation buffer (50 mM Tris-HCl (pH 7.5), 150 mM NaCl, 1% Nonidet P-40, 0.1% SDS, and 1% sodium deoxycholate) supplemented with 1 mM Na3VO4, 1 mM DTT, 1 mM NaF, and protease inhibitor mixture (Roche Applied Science). The samples were boiled for 5 min, and an equal amount of protein was analyzed on SDS-PAGE.

For immunoprecipitation, cells (1 × 10^7^) were lysed in immunoprecipitation buffer (50 mM HEPES, pH 7.6, 150 mM NaCl, 5 mM EDTA, 0.1% Nonidet P-40). After centrifugation (10 min at 15 000 × g) to remove particulate material, supernatants were incubated with antibodies (1:100) against IgG, HSF1, Ku70, Ku86, GST, and DNA-PKcs with constant agitation at 4°C. Immunocomplexes were precipitated with protein A-Sepharose (Sigma) and analyzed by SDS-polyacrylamide gel electrophoresis.

### Irradiation

Cells were exposed to γ-rays using a ^137^Cs γ-ray source (Atomic Energy of Canada) with a dose rate of 3.81 Gy/min.

### Immunofluorescence analysis

Immunofluorescence analysis was performed essentially as previously described (41).

### Determination of HR or NHEJ activity

The experiment was performed as described previously (21). In detail, U2OS cells, stably expressing pimDR-GFP or pimEJ5-GFP (generously gifted from Dr. Jeremy M. Stark, Beckman Research Institute, USA) were transfected with 4 μg of I-SceI (pCB-Asce) with 20 μl of Lipofectamine 2000 (Invitrogen) in 1 ml of OptiMEM(gibco) with 4 μg of Mock vector or Flag-HSF1. Media were changed 3 hours after transfection. The cells were incubated for additional 72 hours and the percentage of GFP positive cells was determined by fluorescence-activated cell sorting (FACS) analysis.

### *In vitro* protein-binding assay

Pull-down assays were performed by incubating the GST-HSF1 fusion proteins, loaded on glutathione-sepharose beads, with cellular lysates in binding buffer, for 18 hours at 4°C. The beads were washed extensively, resuspended in sample buffer, and analyzed by SDS-PAGE and western blotting with the indicated Abs.

### Reverse transcription-polymerase chain reaction (RT-PCR)

RT-PCR was performed essentially as previously described (42).

### Neutral comet assay

The cells were exposed to IR and subjected to a comet assay to detect DNA damage and repair at the single-cell level, using a commercially available assay system (Trevigen).

### Phosphatase treatment

Cell extracts were incubated with 40U of λ phosphatase at 30°C for 30 minutes and then analyzed by western blotting and immunoprecipitation.

### Immunohistochemistry

Tumor slides were deparaffinized and rehydrated using xylene and alcohol. For immunoperoxidase labeling, endogenous peroxidase was blocked with 0.3% H_2_O_2_ in Phosphate-buffered saline (PBS) for 15 min at room temperature. Primary anti-HSF1, anti-Ku70 and anti-Ku86 antibodies were reacted with the tissue for 2 h in a humid chamber at 37°C, washed with PBS for 10 min, and sections were then incubated for 30 min at 37°C with secondary antibody. After additional incubation with streptavidin-horseradish peroxidase for 30 min, immunoreactive sites were visualized using 3,3′-diaminobenzidine (DAB) staining for 5 min. Sections were counterstained with Harris’ hematoxylin, dehydrated, and mounted with coverslips.

### Spontaneous mammary tumor development and histological examination

Spontaneous mammary tumors were induced to female Sprague-Dawley (SD) rats by oral administration of DMBA (15 mg/rat, Sigma). Rats were autopsied under ether anesthesia 26 weeks after DMBA and collected tumors were fixed in 10% neutral buffered formalin, and paraffin-embedded sections were routinely prepared and stained with hematoxylin and eosin (H&E) for histological evaluation. The grading of rat breast carcinomas parallels the efforts in human breast carcinoma to establish a basis for the estimation of virulence and thus probable prognosis. In rat tumors, the grading is mainly based on histology, since unlike human tumors, other criteria such as metastases to lymph nodes and distant organs are exceedingly rare. The major criteria are irregularities in the size, shape and staining of cells and nuclei, and the frequency of mitoses. Other findings, such as necrosis, fibrosis and hemorrhage, also influence the grading (22). The studies were conducted under guidelines for the use and care of laboratory animals and were approved by the Institutional Animal Care and Use Committee of the Korean Institute of Radiological and Medical Sciences (KIRAMS).

### Statistical analysis

All statistical significance was determined by the Student's *t*-test. The differences were considered significant if the *p*-value was less than 0.05.

## SUPPLEMENTARY DATA FIGURES


