# Supplementary material for: Heat shock factor 1, an inhibitor of non-homologous end joining repair
Source: Oncotarget. 2015 Aug 24;6(30):29712–24. doi: 10.18632/oncotarget.5073 (PMC4745757; doi:10.18632/oncotarget.5073)
Supplement: Supplementary file 1 [file oncotarget-06-29712-s001.pdf]

SUPPLEMENTARY DATA

Reagents

Cleaved caspase-3 and cleaved PARP1 were obtained from Cell Signaling Technology.

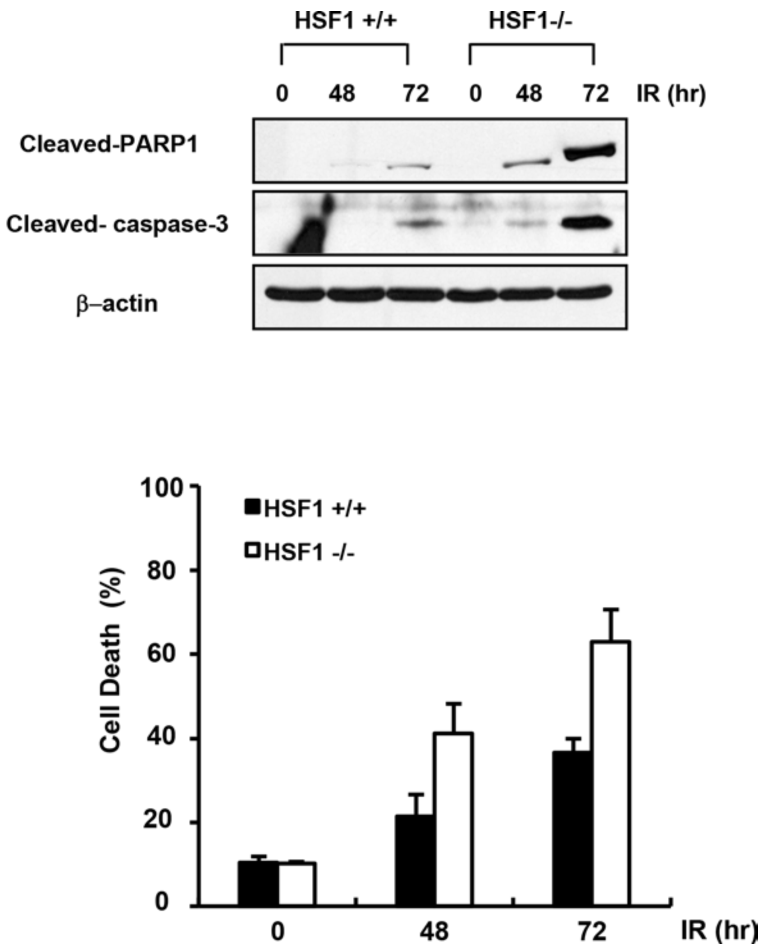

Supplementary Figure S1: HSF1 effects on IR-mediated cell death. After radiation (IR, 10 Gy) was applied to HSF1 wild type (HSF1+/+) or knockout (HSF1−/−) MEF cells, cell death was analyzed by western blotting (upper) and FACS (bottom). Each data point represents the mean ± SE of three experiments.

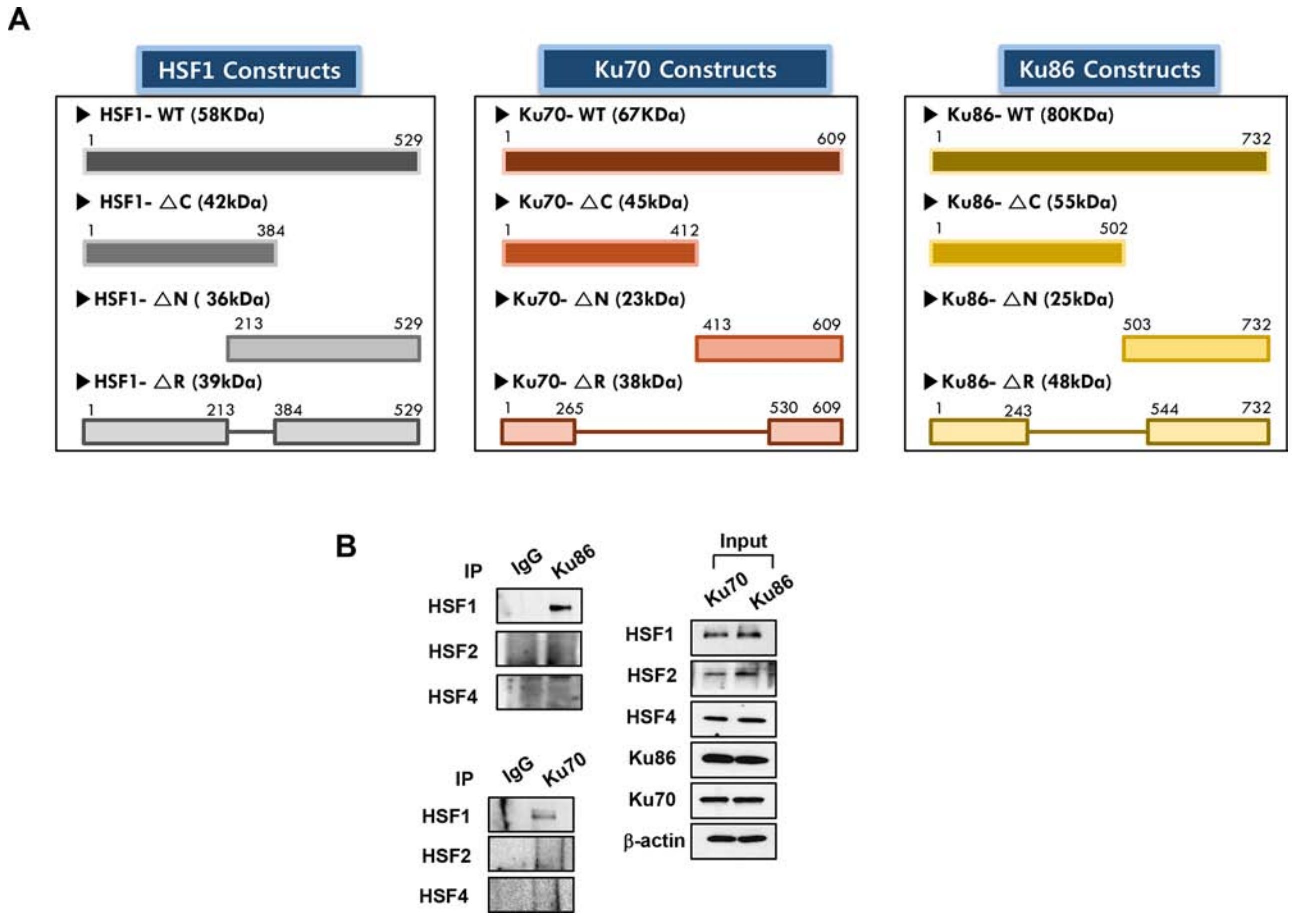

Supplementary Figure S2: Binding activity of other HSFs with Ku70 or Ku86. A. Various deletion constructs of Flag-tagged HSF1, Ku70 and Ku86. **B.** Western blotting or immunoblotting was performed following immunoprecipitation using HOS cell extracts.

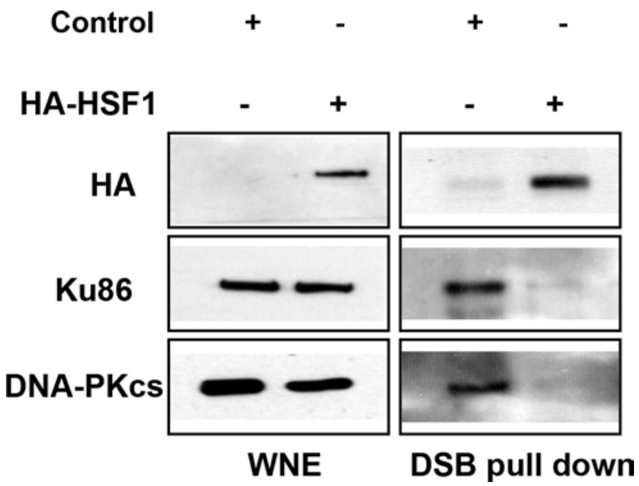

Supplementary Figure S3: Control or HA-HSF1-transfected HOS cells were harvested. The protein levels of Ku86 and DNA-PKcs in the dsDNA pull-down lysates, as well as in whole nuclear extracts (WNE), were analyzed.

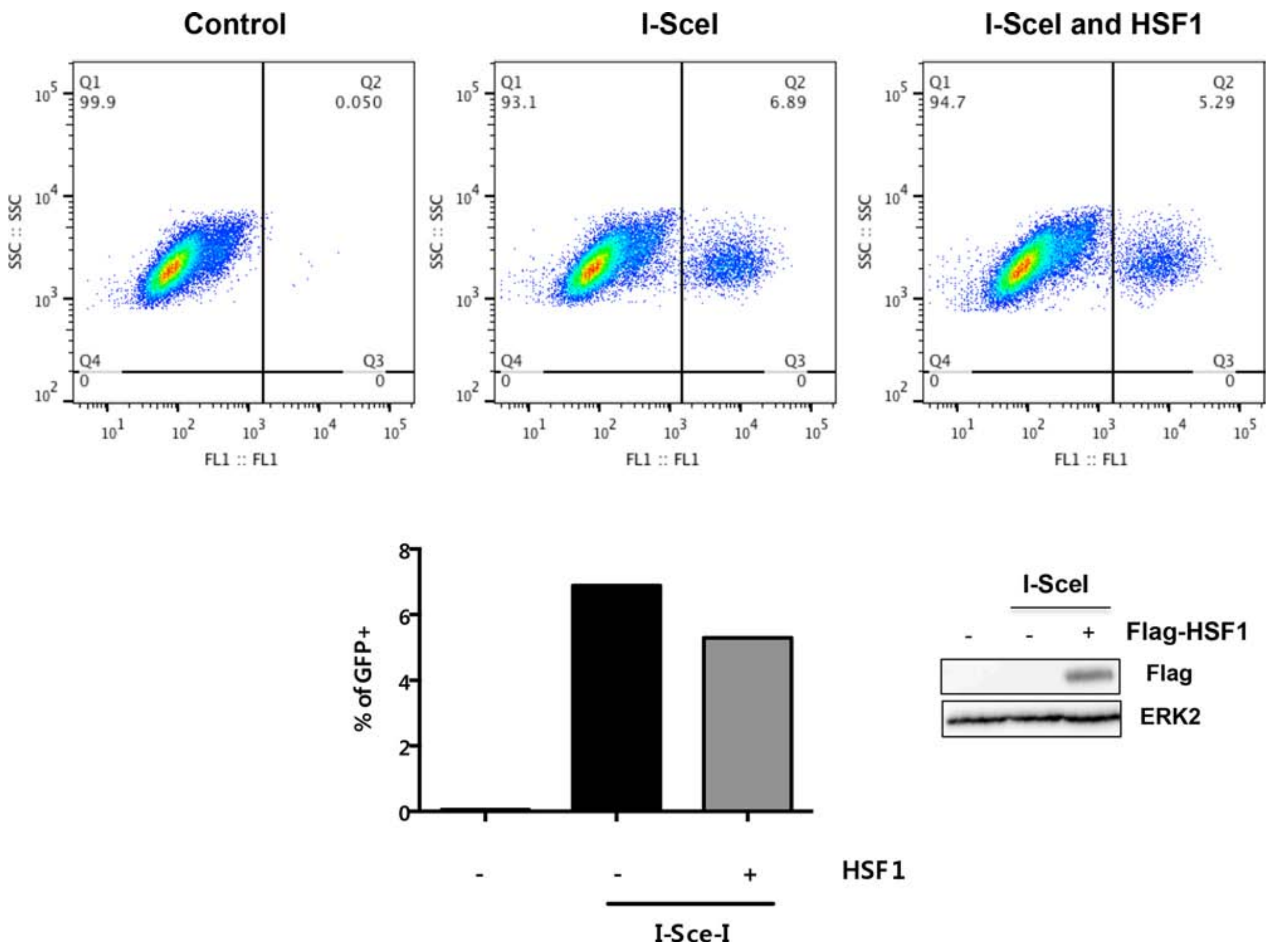

Supplementary Figure S4: GFP+ cells with mock or vector expression in U2OS cells stably expressing DR-GFP were determined by FACS analysis. GFP+ cells were graphically presented. HSF1 expression level was determined by immunoblotting with Flag. ERK2 was used for equal loading control.

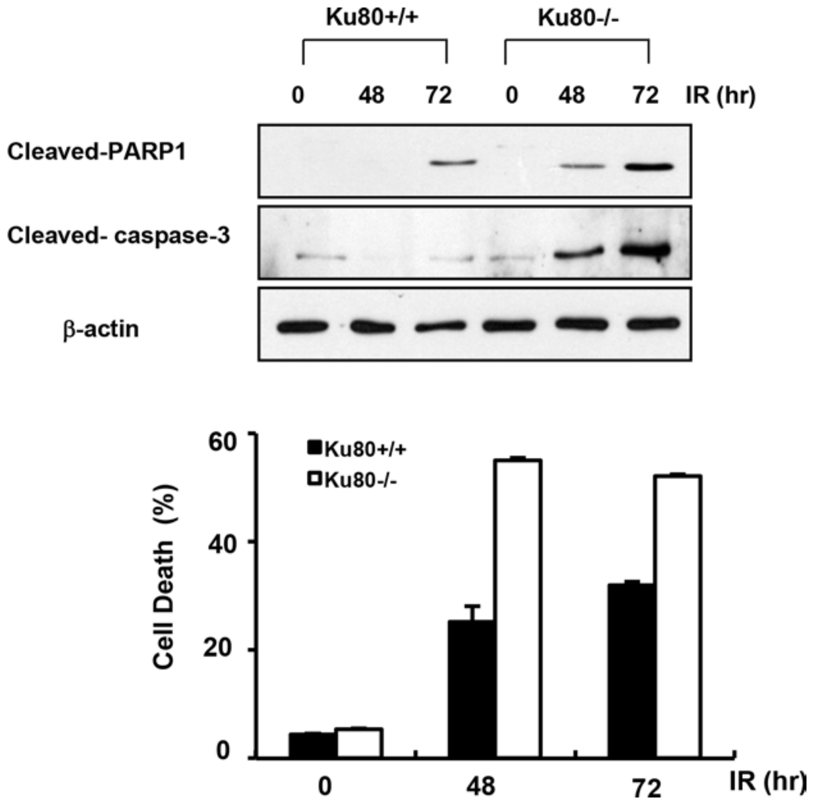

Supplementary Figure S5: Effects of Ku80 on IR-mediated cell death. After of 10 Gy of radiation (IR) was applied Ku80 defective cells, cell death was analyzed by western blotting (upper) and FACS (bottom). Each data point represents the mean ± SE of three experiments.

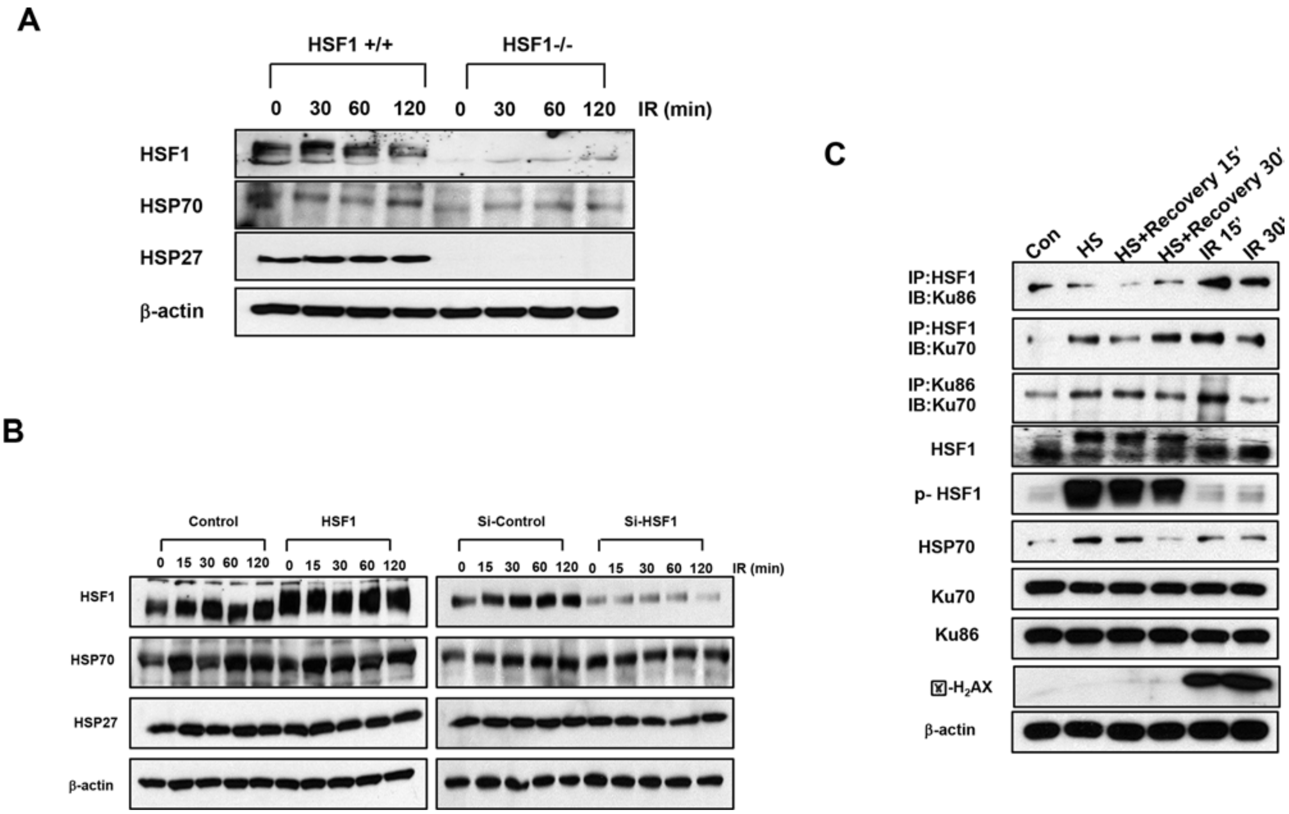

Supplementary Figure S6: Effects of HSF1 transcriptional activity on binding activity of HSF1-Ku86 or HSF1-Ku70. Western blotting or IB was performed using HSF1 wild type (HSF1+/+) or knockout (HSF1−/−) MEF cells **A.** or Si-HSF1 or Flag-HSF1-transfected HOS cell extracts **B.** was conducted at the indicated time points following treatment of cells with an IR dose of 5 Gy and 10 Gy. **C.** HSP70 stably transfected NCI-H460 lung cancer cells were heat shocked (HS) for 30 min at 42°C followed by indicated recovery times or exposed IR (10 Gy), total proteins were analyzed for Western blotting or immunoprecipitation.

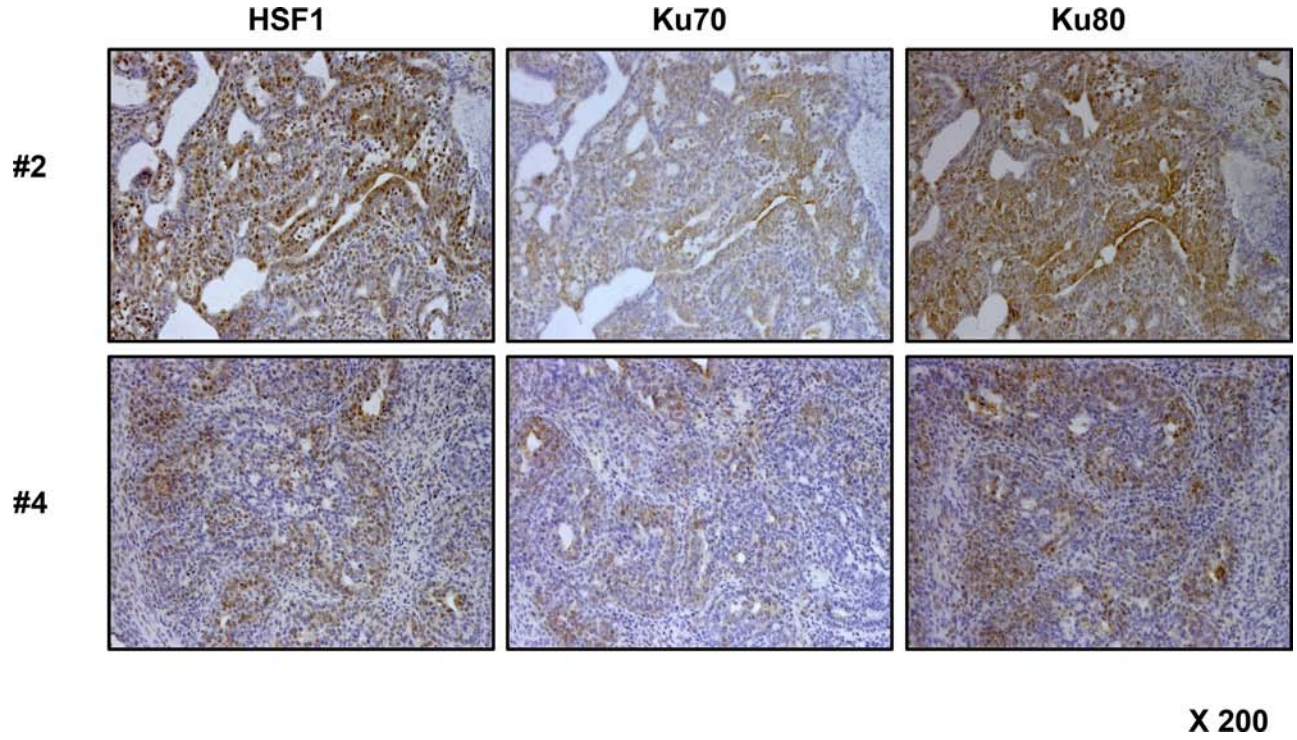

Supplementary Figure S7: Immunohistochemistry for detection of HSF1, Ku70 and Ku80 in rat mammary tumors. Tumor sections (magnification × 200) from rat mammary tumors (#2 and #4) were analyzed by immunohistochemistry for detection of HSF1, Ku70 and Ku80 (brown staining).
